# International Evidence-Based Medicine Survey of the Veterinary Profession: Information Sources Used by Veterinarians

**DOI:** 10.1371/journal.pone.0159732

**Published:** 2016-07-26

**Authors:** Selene J. Huntley, Rachel S. Dean, Andrew Massey, Marnie L. Brennan

**Affiliations:** Centre for Evidence-based Veterinary Medicine, School of Veterinary Medicine and Science, The University of Nottingham, Sutton Bonington Campus, Loughborough, Leicestershire, United Kingdom; Ross University School of Veterinary Medicine, SAINT KITTS AND NEVIS

## Abstract

Veterinarians are encouraged to use evidence to inform their practice, but it is unknown what resources (e.g. journals, electronic sources) are accessed by them globally. Understanding the key places veterinarians seek information can inform where new clinically relevant evidence should most effectively be placed. An international survey was conducted to gain understanding of how veterinary information is accessed by veterinarians worldwide. There were 2137 useable responses to the questionnaire from veterinarians in 78 countries. The majority of respondents (n = 1835/2137, 85.9%) undertook clinical work and worked in a high income country (n = 1576/1762, 89.4%). Respondents heard about the survey via national veterinary organisations or regulatory bodies (31.5%), online veterinary forums and websites (22.7%), regional, discipline-based or international veterinary organisations (22.7%) or by direct invitation from the researchers or via friends, colleagues or social media (7.6%). Clinicians and non-clinicians reportedly used journals most commonly (65.8%, n = 1207/1835; 75.6%, n = 216/286) followed by electronic resources (58.7%, n = 1077/1835; 55.9%, n = 160/286), respectively. Respondents listed a total of 518 journals and 567 electronic sources that they read. Differences in veterinarian preference for resources in developed, and developing countries, were found. The nominated journals most read were the Journal of the American Veterinary Medical Association (12.7% of nominations) for clinicians and the Veterinary Record (5.7%) for non-clinicians. The most accessed electronic resource reported was the Veterinary Information Network (25.6%) for clinicians and PubMed (7.4%) for non-clinicians. In conclusion, a wide array of journals and electronic resources appear to be accessed by veterinarians worldwide. Veterinary organisations appear to play an important role in global communication and outreach to veterinarians and consideration should be given to how these channels could be best utilised for effective dissemination of key research findings.

## Introduction

Veterinarians across the world play an important societal role in the safeguarding of animal and public health [1, 2]. There is disparity in veterinary information sources across the globe [3] and there is less access to information technology systems in developing countries [4]. However, with continuing advances in technology, the internet and increased accessibility to electronic media worldwide [5], a wider array of resources for veterinary information are becoming more available to more people.

It is the responsibility of veterinarians to produce, interpret, communicate, and apply scientific information in the best possible way to make informed decisions and take adequate actions in the care of animals, however, obstacles to this process still exist [6]. Much of the content of the unprecedented breadth of resources now available are not peer-reviewed, and peer-reviewed publications can vary in quality and strength of evidence [7–9]. Therefore it is important that veterinarians are equipped with the tools to make judgements about the quality of the evidence available, and to habitualise themselves in the application of these techniques when encountering new information. Evidence-based veterinary medicine (EVM] is defined as “the use of best relevant evidence in conjunction with clinical expertise to make the best possible decision about a veterinary patient. The circumstances of each patient, and the circumstances and values of the owner/carer, must also be considered when making an evidence-based decision” [10, 11]. Thus, in addition to having access to robust information resources, it is also important that veterinarians are not only practiced in the methodologies of assessing the quality of the information available to optimise their decision-making [12, 13], but to use these skills alongside their clinical judgement and experience.

Information resources accessed by the veterinary profession in the United Kingdom have previously been identified [14]. However, there have been no previous peer-reviewed studies assessing how veterinarians in different countries source veterinary information. The aim of this study was to describe the current breadth of veterinary resources used by veterinarians internationally, to determine which ones are perceived as useful and understand how they are accessed. Additionally, the aim was to determine the most successful way to contact veterinarians globally. This knowledge can be used to help understand the best ways to deliver relevant information to the international veterinary community to enhance the use of evidence by veterinarians worldwide.

## Materials and Methods

### Study design and delivery

An international survey of the veterinary profession about evidence-based veterinary medicine was conducted in 2011 via an online questionnaire. The questionnaire aimed to collect information about the demographics and place and type of employment of respondents and asked a number of questions across 4 main sections. Questions were almost identical to those asked and reported in a similar survey of UK based veterinary professionals [14, 15]. Information was sought from respondents about whether they had heard of the term EVM, together with the resources that they used to access veterinary information. Those who did some clinical work were also asked about sources of veterinary information accessed by their clients and the common species and conditions which were most commonly encountered in practice. Additionally, questions were asked on opinions of respondents on participation in practice based research. There were 53 questions in the questionnaire, consisting of both open and closed questions. This paper reports the demographics of the target veterinary population, including the clinical and geographical background of respondents and highlights results regarding the questionnaire sections relating to the resources that veterinarians use to access veterinary information. Results from other aspects of the questionnaire will be reported in other publications. Open questions focused on which information sources were accessed and respondents were required to list up to 10 journals and up to 10 electronic resources. Respondents were also asked, as open questions, to nominate a single journal and a single electronic resource that they found most useful for obtaining veterinary information. Questions asking respondents which country they worked and trained in, were also open.

Several resources were used to assist in the creation of questions which resulted in the minimisation of ambiguity and optimisation of clarity [16–18]. The questionnaire was both pre-tested and piloted [19, 20]. The pre-test was carried out by five members of the Centre of Evidence–based Veterinary Medicine in the School of Veterinary Medicine and Science, University of Nottingham. Following changes from the pre-test, a pilot was carried out with 18 veterinarians based in practice, industry, academia and those who were self-employed. Pilot participants originated from Denmark, The Netherlands, USA, Australia, Spain, India, France, Chile and Switzerland. The questionnaire was constructed using software provided by Cvent (2011 Cvent Inc.), an online survey company. The software had the ability for logic to be incorporated into the design of the survey. Logic allowed ‘funnelling’ of individuals through the questionnaire process by the exclusion of irrelevant questions, reducing completion times and the likelihood of non-response bias.

The target population was all veterinarians working outside of the UK; there was no definitive global list of veterinarians or organisations available. Veterinary organisations were contacted initially using a list of international veterinary groups from Appendix 1 of section 4 of the 2010 Royal College of Veterinary Surgeons (RCVS) Register of Members [21]. These organisations were contacted by email, where an email address was available. If an email address was not available or the first email attempt bounced, an internet search for the veterinary organisation was carried out, and further email addresses found where possible. The authors made up to three attempts to identify contact details for each organisation. During these additional searches, email addresses for organisations that were considered of relevance but had not yet been contacted were identified and emails sent to these organisations. Organisations that did not have an email address but had a website and a contact page were contacted by copying a standard piece of text about the study into the contact box. For organisations where email addresses could not be found but a fax number was available, faxes were sent.

Additionally, a snowball sampling approach was also taken whereby individuals within the Centre for Evidence-based Veterinary Medicine and academic members of staff at the School of Veterinary Medicine and Science at the University of Nottingham were asked to share details for any international veterinary organisations, listserves or chat sites, or personal contacts that they felt might be relevant to this study. The organisations identified were contacted directly, or on behalf of the final author (MB). Managers of online veterinary sites and further listserves deemed to be veterinary related (e.g. International Veterinary Information Service—IVIS) were also contacted. All of these contacts (with the exception of the online veterinary sites) were emailed a seeding email and were then sent the link to the survey between 1.5–3 weeks later. A reminder was then sent 3–5 weeks after the link was sent. Emails were sent between June and September 2011.

### Collation and analysis of data

Data from the online survey were collated and stored securely by Cvent (Cvent Inc. 2011), after which they were downloaded and stored in a Microsoft Excel spreadsheet (Microsoft, 2007). Responses that were classified as unusable were excluded from the analysis. These exclusions were respondents that had only completed the demographic information and had left the majority of the remaining fields blank, responses that appeared to be duplicates from the same IP address with identical answers who were assumed to be the same respondents, and those working in the UK.

The respondents’ countries of work and training were classified into one of 12 classifications of continent and subcontinent using United Nations Classification [22] and into five categories of development according to the International Monetary Fund (IMF) Classification (high income, upper middle income, middle income, lower middle income and low income countries; [23]). Clinicians were defined as veterinarians who reported they spent at least 1% of their time doing some clinical work, and non-clinicians as those who reported that they did less than 1% of clinical work. Work classified as ‘other’ small animal pet work was defined as pets that were not dogs, cats and rabbits; namely pet rodents, guinea pigs, ferrets, reptiles, birds and fish.

Veterinary information sources nominated by respondents were verified by a list compiled from recent research into the coverage of veterinary journals by bibliographic databases [24]. Those that were not found were checked against other available lists of journal resources (scimagojr.com; UlrichsWeb) and then were searched via Google. From these sources, a template of journal titles and electronic source titles was created. Using this template, all veterinary information sources that were mentioned by respondents were coded for analysis. Descriptive analyses were performed in Microsoft Excel (Microsoft, 2007), chi-squared analysis and Wilcoxon Mann-Whitney test performed in Stata 13 [25] and z-tests for the differences in proportions were performed using an online epidemiological tool (http://epitools.ausvet.com.au). Significance level was set at p<0.05. This project received ethical approval by the Ethics Committee at the School of Veterinary Medicine and Science at The University of Nottingham. As not all respondents answered all questions, denominators are stated where appropriate.

## Results

There were 220 organisations identified from the RCVS register across 111 Countries. Of these 220 organisations, 113 emails were deemed successful (the email was sent and no error message was received), 48 were deemed not successful (emails bounced) and no email was given for 59 organisations. An internet search of those 48 organisations for which the initial contact email from the RCVS register bounced yielded 44 available contacts, of which there were 34 that were deemed successful (31 email successes and 3 web contact page successes) and 10 that were not successful (9 emails bounced and 1 web page contact failed). An internet search for a contact for each of those 59 organisations without an email address yielded 45 contacts, of which 28 were deemed successful (21 emails, 3 web contact pages and 4 faxes sent) and 17 were not successful (13 emails bounced, 3 web contact page failed and 1 fax failed to send). Fifty one contacts were also added either by ad hoc identification of organisations thought to be relevant or via the snowball approach. A total of 226 successful (as defined above) contacts were therefore made.

### Respondent demographics

There were 2422 replies to the questionnaire from 79 countries, of which 2137 from 78 countries were usable. Of the 285 unusable responses, there were 251 responses where respondents had answered demographic information only, 19 responses that appeared to be duplicates from the same IP address with identical answers and 15 respondents who were working in the UK. There were 1835 clinicians and 286 non-clinicians in the usable replies.

General demographic information collected suggested respondents (n = 2137) were generally older and were likely to be clinicians (Table 1).

**Table 1:** General respondent demographic information.

**Age**	**Median 43 years**	**IQR 34–52 years**
**Years qualified**	Median 16 years	Range 0–53 years
Gender*	Female n = 1242/2127 (58.2%)	Male n = 885/2127 (41.3%)
**Work type**^$^	Clinicians n = 1835/2121 (85.9%)	Non-clinicians n = 286/2121 (13.4%)
**Country of work (clinicians only)**^@^	Developed n = 1576/1762 (89.4%)	Developing n = 186/1762 (10.6%)

* Not declared n = 10/2137 (0.5%)

^$^ Not declared n = 16/2137 (0.7%)

^@^ Not declared n = 68/1835 (3.7%); working in multiple countries n = 5/1835 (0.3%).

The majority of clinicians (n = 1767/1835; 96.3%) stated their country of work; this information was not collected from non-clinicians. There were 78 countries represented and five respondents stated that they worked in multiple countries (therefore n = 1762). The majority of respondents worked in a high income country (n = 1576/1762, 89.4%), with 131 respondents (7.4%) working in upper middle income countries, 47 respondents (2.7%) working in lower middle income countries and 8 respondents (0.5%) working in low income countries. The majority were working in North America (n = 717, 40.7%) and Europe (n = 622, 35.3%), with fewer working in Oceania (n = 184, 10.4%), Africa (n = 121, 6.9%) and the Far East and Asia (n = 53, 3.0%) (Table 2). The top five most represented countries of work were USA (606 respondents, 34.4%), Sweden (206 respondents, 11.7%), Australia (151 respondents, 8.6%), Canada (110 respondents, 6.2%) and South Africa (101 respondents, 5.7%). Respondents also reported their country of veterinary training; this profile was very similar to that of country of work.

**Table 2:** Respondents place of work, according to the UN classification of continent and country. Developed countries were those considered high income in IMF classification (n = 1576), and developing countries were those belonging to the remaining 4 IMF classification categories (n = 186). Only data from clinician respondents were collected.

**Africa**					
**Eastern Africa**					
Seychelles*	1	Uganda*	2	Zambia*	1
**Middle and Southern Africa**					
Cameroon*	1	South Africa*	101		
**Northern Africa**					
Egypt*	1	Sudan*	3	Tunisia*	1
**Western Africa**					
Burkina Faso*	1	Nigeria*	9	Senegal*	1
**Americas**					
**Caribbean**					
Cuba*	1	Dominica*	1	Jamaica*	2
Puerto Rico	18	Trinidad and Tobago	8		
**Central America**					
Belize*	1	Costa Rica*	2	Mexico*	1
Panama*	1				
**North America**					
Bermuda	1	Canada	110	USA	606
**South America**					
Argentina*	1	Brazil*	6	Chile	2
Uruguay	5				
**Asia**					
**Eastern and South-Eastern Asia**					
China*	1	Hong Kong^$^	11	Japan	5
Malaysia*	1	Singapore	5		
**Southern Asia**					
Afghanistan*	1	Bangladesh*	3	India*	13
Nepal*	1	Pakistan*	5	Sri Lanka*	8
**Western Asia**					
Bahrain	1	Iraq*	1	Israel	8
Kuwait	1	Turkey*	2	United Arab Emirates	2
**Europe**					
**Eastern Europe**					
Czech Republic	1	Poland	1	Romania*	1
Russia	1				
**Northern Europe**					
Denmark	14	Estonia	4	Finland	43
Iceland	3	Ireland	53	Latvia	5
Norway	9	Sweden	206		
**Southern Europe**					
Bosnia and Herzegovina*	3	Croatia	1	Greece	4
Italy	17	Kosovo*	5	Macedonia*	1
Portugal	3	Serbia*	2	Slovenia	1
Spain	21				
**Western Europe**					
Austria	63	Belgium	6	France	50
Germany	64	Liechtenstein	1	Luxembourg	9
Netherlands	18	Switzerland	11		
**Oceania**					
**Australia and New Zealand, Melanesia and Polynesia**					
Australia	151	Cook Islands	1	New Caledonia^@^	1
New Zealand	31				

*Developing countries

^@^Overseas territory

^$^ Special administrative region.

### Respondent work type

Most clinicians (n = 1525/1835; 83.1%) nominated that they worked in private clinics and for the majority this was the sole type of work they did (1240/1525, 81.3%). Other common work categories of all respondents were university clinical and/or teaching work (n = 308/2137, 14.4%) and work in research in a university or institute (n = 289/2137, 13.5%; Table 3).

**Table 3:** Work type and combinations of work types of all respondents (n = 2137).

Work type	All	%	Male	%	Female	%	Work combination	n	%
	(n = 2137)		(n = 885)		(n = 1242)				
Private clinic/s*	1566	73.3	590	66.7	968	77.9	Private clinic only	1240	58.0
University clinic or education*	308	14.4	160	18.1	146	11.8	Private clinic and other combination	326	15.3
Research (university or institute)^@^	289	13.5	140	15.8	147	11.8	University clinic or research with some other combination	295	13.8
Government^$^	191	8.9	104	11.8	87	7.0	Government and other combination	98	4.6
Other	125	5.8	61	6.9	63	5.1	Government only	93	4.4
Animal charity*	107	5	24	2.7	82	6.6	University clinic/education and Research	89	4.2
Public health*	92	4.3	57	6.4	35	2.8	University clinic/education only	84	3.9
Industry *	84	3.9	57	6.4	27	2.2	Research only	59	2.8
Pathology/clinical path lab^@^	45	2.1	27	3.1	18	1.4	Industry only	39	1.8
Outside vet profession	39	1.8	19	2.1	19	1.5	Public health or PH combo but not private clinic	26	1.2
Taking a career break	12	0.6	2	0.2	10	0.8	Charity only	13	0.6
Army	6	0.3	4	0.5	2	0.2	Pathology/clinical path lab or pathology and industry only	8	0.4

Association between work type and gender

*p>0.001,

^@^p = 0.008,

^$^p = 0.001 (z-test for difference in two proportions).

Overall, the majority of respondents worked in one work category only (n = 1598, 74.8%) and a smaller number in two (n = 389, 18.2%), three (n = 118, 5.5%) or four or more (n = 29, 1.4%) category combinations. Clinicians who worked in developing countries were more likely to work in more than one category (n = 74/186, 39.8%) than those in developed countries (n = 325/1576, 20.6%; p>0.0001). A larger proportion of non-clinicians (66.1%, n = 189/286) than clinicians (38.2%, n = 701/1835; p>0.0001) held a further veterinary qualification in addition to their veterinary degrees.

The majority of clinicians stated that they spent most of their time doing clinical work (mode 90% of working time, IQR 70–100%). Clinicians in developed countries were significantly more likely to work in private practice as their sole type of work (n = 1106/1576, 70.2%) than those in developing countries (n = 75/186, 40.3%; p<0.0001). Clinicians in developing countries were more likely to do some Government (n = 32/186, 17.2%) work than clinicians in developed countries (n = 83/1576, 5.3%; p<0.0001) or a combination of University and Research (n = 27/186, 14.5%) than clinicians in developed countries (n = 67/1576, 4.2%; <0.0001). Most clinicians (63.0%, n = 1157) nominated that they had a personal caseload that was a mixture of first opinion and second opinion work. However 571 (31.1%) had a caseload of first opinion only, 69 (3.8%) had a caseload of second opinion only and 38 (2.1%) had a personal caseload of the category “other”.

The most commonly nominated type of clinical work was with small animals only (dogs, cats, rabbits) for veterinarians working in both developed (468/1576 respondents, 29.7%) and developing countries (38/186, 20.4%). Clinical work comprising a combination of small animals with “other” small animal pet work was also common (developed countries, 320/1576, 20.3%; developing countries, 22/186, 11.8%). The third most common type of clinical work for those in developed countries was equine work only (119/1576 respondents, 7.5%), whilst for those in developing countries this was general production animals (n = 8/186, 4.3%), closely followed by pigs and poultry (n = 7/186, 3.7%).

### How respondents heard about the questionnaire

Over half (n = 1159/2137, 54.2%) of respondents had heard about the questionnaire via a veterinary organisation, whether that was a national organisation, board or regulatory body (n = 674/2137, 31.5%) or other type of veterinary organisation (regional, by discipline and/or international group; n = 485/2137, 22.7%). Overall there were equal numbers of respondents that had heard about the questionnaire via an email periodical, forum or non-specified web source as had heard via regional, by discipline and/or international group veterinary organisation (n = 485/2137, 22.7%). By comparison, fewer respondents had heard about the questionnaire via other methods, such as non-specified email (n = 209/2137, 9.8%), vet schools or other research institutes (n = 49/2137, 2.3%). Direct invitation from the researchers or via friends, colleagues or social media accounted for a small proportion of responses (n = 162/2137, 7.6%) as did those where the respondents listed other sources (n = 32/2137, 1.5%) or didn’t know the source (n = 8/2137, 0.4%).

**Fig 1:**
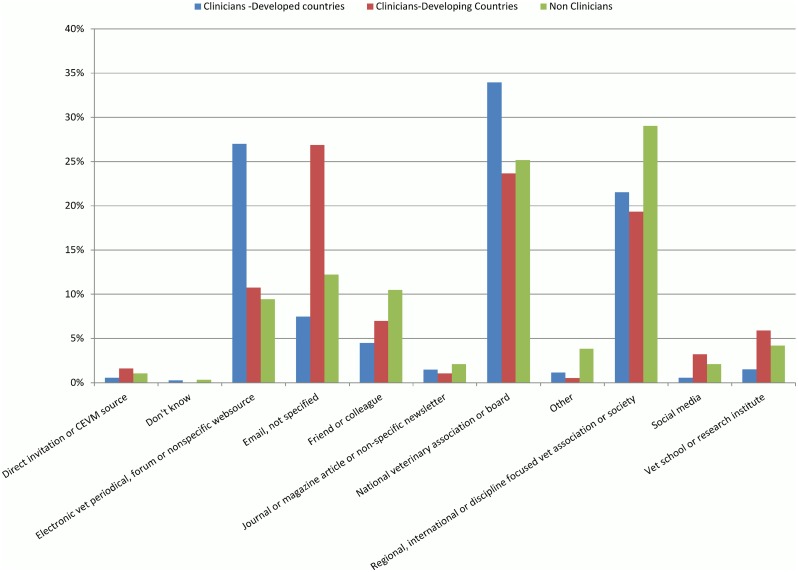
Source of where clinicians working in developed (n = 186) and developing countries (n = 1576), and non-clinicians (n = 286), had heard about the international questionnaire.

There were differences between how clinicians and non-clinicians stated they had heard about the questionnaire (Fig 1). Overall, clinicians were more likely than non-clinicians to have heard about the questionnaire from a national veterinary organisation or regulatory board (clinicians n = 595/1835, 32.4%; non-clinicians n = 72/286, 25.2%; p = 0.011). Non-clinicians were more likely than clinicians to have heard about the questionnaire from another type of veterinary regional, discipline and/or international organisation (non-clinicians n = 83/286, 29.0%; clinicians n = 396/1835, 21.6%; p = 0.004). Amongst clinicians, those working in developing countries were most likely to have heard about the questionnaire from a non-specified email source (n = 50/186, 26.9%).

### Sources of veterinary information for veterinarians

#### Journal and electronic resources that respondents accessed

Respondents listed a total of 518 journals and 567 electronic resources they accessed (n = 1423/2137 respondents). Seven hundred and fourteen (33.4% of 2137) respondents did not list any journal or electronic resources.

The majority of clinicians (n = 1207/1835, 65.8%, median 3 journals, IQR 2–5) and non-clinicians (n = 216/286, 75.5%, median 4 journals, IQR 3–6) stated using journals as sources of veterinary information. The three journals with the most nominations as being read by respondents were Journal of the Veterinary Medical Association (JAVMA) (n = 626/5559, 11.3%), Veterinary Medicine (n = 226/5559, 4.1%), and the Journal of Veterinary Internal Medicine (n = 165/5559, 3.0%; Table 4). However, the most nominated journals were slightly different between groups by work type; for clinicians JAVMA (n = 581/4557, 12.7%), Veterinary Medicine (n = 221/4557, 4.8%) and Compendium (n = 161/4557, 3.5%) were common; for non-clinicians Veterinary Record (n = 57/1002, 5.7%), Preventive Veterinary Medicine (n = 52/1002, 5.2%) and JAVMA (n = 45/1002, 4.5%) were common (Table 5). The most commonly read journals as nominated by respondents were also different by development status of country of work; for clinicians working in developing countries, the most commonly read journal was the Journal of the South African Veterinary Association (n = 37/387, 9.6%; Table 4). When respondents from the USA were removed, the most read journal nominated by clinicians remained JAVMA (n = 144/2641; 5.5%), followed by the Australian Veterinary Journal (n = 99/2641; 3.7%) and Svensk Veterinärtidning (n = 94/2641; 3/6%). For non-clinicians, the list of journals remained the same.

**Table 4:** Table of the top ten journals most read as nominated by respondents working in developing and developed countries. Respondents could nominate up to 10 different journals.

Rank	Developing	n	%	Developed	n	%	Country not stated	n	%	Overall	n	%
	(387 responses)			(4134 responses)			(1038 responses)^$^			(5559 responses)		
1	Journal of the South African Veterinary Association	37	9.6	Journal of the American Veterinary Medical Association	558	13.5	Veterinary Record	59	5.7	Journal of the American Veterinary Medical Association	626	11.3
2	Veterinary Medicine*	25	6.5	Veterinary Medicine*	194	4.7	Preventive Veterinary Medicine*	52	5.0	Veterinary Medicine*	226	4.1
3	Journal of the American Veterinary Medical Association	18	4.7	Clinician's Brief*	158	3.8	Journal of the American Veterinary Medical Association	50	4.8	Journal of Veterinary Internal Medicine	165	3.0
4	Vet News	14	3.6	Compendium: Continuing Education For Veterinarians*	148	3.6	Veterinary Pathology*	35	3.4	Compendium: Continuing Education For Veterinarians*	164	3.0
5	Veterinary Clinics of North America	13	3.4	Journal of Veterinary Internal Medicine	140	3.4	Australian Veterinary Journal	20	1.9	Clinician's Brief*	163	2.9
= 5	Veterinary Record	13	3.4				Veterinary Microbiology*	20	1.9			
6	Compendium: Continuing Education For Veterinarians*	12	3.1	Journal of the American Animal Hospital Association	125	3.0	American Journal of Veterinary Research	19	1.8	Journal of the American Animal Hospital Association	142	2.6
= 6							Veterinary Research*	19	1.8			
7	Journal of Small Animal Practice*	9	2.3	DVM 360 magazine	121	2.9	Emerging Infectious Diseases*	18	1.7	Veterinary Record	132	2.4
= 7	Journal of Veterinary Internal Medicine	9	2.3									
8	In Practice	8	2.1	Australian Veterinary Journal	97	2.3	Journal of Veterinary Diagnostic Investigation*	17	1.6	DVM 360 magazine	126	2.3
= 8				Svensk Veterinärtidning	94	2.3						
9	American Journal of Veterinary Research	7	1.8	Equine Veterinary Journal	92	2.2	Journal of Comparative Pathology*	16	1.5	Australian Veterinary Journal	121	2.2
= 9	Indian Veterinary Journal						Journal of Veterinary Internal Medicine	16	1.5			
= 9	Journal of the American Animal Hospital Association	7	1.8									
10	Equine Veterinary Journal	6	1.6	Veterinary Economics*	91	2.2	Journal of Dairy Science*	13	1.3	American Journal of Veterinary Research	106	1.9
= 10	The Veterinary Journal*	6	1.6				The Veterinary Journal*	13	1.3	Equine Veterinary Journal	106	1.9
= 10	Veterinary Parasitology*	6	1.6							Journal of Small Animal Practice*	106	1.9

^$^1002 of 1038 (96.5%) responses where country was not stated were responses by non-clinicians. Non-clinicians were not asked country of work in the questionnaire.

*International journal.

**Table 5:** Top ten journals most read as nominated by clinicians and non-clinicians. Respondents could nominate up to 10 different journals.

Rank	Clinicians	n	%	Non-Clinicians	n	%
	(4557 responses)			(1002 responses)		
1	Journal of the American Veterinary Medical Association	581	12.7	Veterinary Record	57	5.7
2	Veterinary Medicine*	221	4.8	Preventive Veterinary Medicine*	52	5.2
3	Compendium: Continuing Education For Veterinarians*	161	3.5	Journal of the American Veterinary Medical Association	45	4.5
= 3	Clinician's Brief*	158	3.5			
4	Journal of Veterinary Internal Medicine	150	3.3	Veterinary Pathology*	35	3.5
5	Journal of the American Animal Hospital Association	133	2.9	Australian Veterinary Journal	20	2
= 5				Veterinary Microbiology*	20	2
6	DVM 360 magazine	123	2.7	Veterinary Research*	19	1.9
7	Australian Veterinary Journal	101	2.2	Emerging Infectious Diseases*	18	1.8
= 7	Equine Veterinary Journal	99	2.2			
8	Journal of Small Animal Practice*	95	2.1	Journal of Veterinary Diagnostic Investigation*	17	1.7
9	Journal of Feline Medicine and Surgery*	94	2.1	Journal of Comparative Pathology*	16	1.6
10	Svensk Veterinärtidning	94	2.1	American Journal of Veterinary Research	15	1.5
= 10	Veterinary Clinics of North America	94	2.1	Journal of Veterinary Internal Medicine	15	1.5

*International journal.

The percentage of respondents nominating that they used electronic resources was slightly smaller than those reading journals (58.7% of clinicians, n = 1077/1835, median = 2 e-resources, 1QR 1–3; 55.9% of non-clinicians n = 160/286, median = 3 e-resources, IQR = 1–4). The Veterinary Information Network (VIN) received just under a quarter (n = 671/3047, 22.0%) of nominations as a top ten most accessed electronic resource (Table 6) The top three most read electronic resources overall by clinicians in developed and developing countries were VIN, IVIS and PubMed (Table 6). For non-clinicians, the top 3 were PubMed, University websites or libraries and the OIE website (Table 7).

**Table 6:** Table of the top ten electronic resources most accessed as nominated as by respondents working in developing and developed countries. Respondents could nominate up to 10 electronic resources.

Rank	Developing	n	%	Developed	n	%	Country not stated	n	%	Overall	n	%
	(207 responses)			(2317 responses)			(523 responses)^@^			(3047 responses)		
**1**	IVIS	26	12.6	VIN	630	27.2	PubMed	40	7.6	VIN	671	22.0
**2**	VIN	17	8.2	IVIS	147	6.3	University Websites or library	24	4.6	IVIS	191	6.3
**= 2**							VIN	24	4.6			
**3**	PubMed	14	6.8	PubMed	121	5.2	OIE	23	4.4	PubMed	175	5.7
**4**	Google	11	5.3	University Websites or library	88	3.8	IVIS	18	3.4	University Websites or library	121	4.0
**5**	University Websites or library	9	4.3	Dvm360	57	2.5	ProMed	14	2.7	American Veterinary Medical Association	73	2.4
**6**	South African Veterinary Association Ruralvet Chat group	7	3.4	American Veterinary Medical Association (AVMA)	55	2.4	American Veterinary Medical Association (AVMA)	13	2.5	Dvm360	63	2.1
**7**	Merck Veterinary Manual	6	2.9	American Association of Equine Practitioners (AAEP) listserve	49	2.1	Food and Agriculture Organisation (FAO)	11	2.1	Google	60	2.0
**= 7**							Google	11	2.1			
**8**	American Veterinary Medical Association	5	2.4	Google	38	1.6	Cornell UCVM^$^	9	1.7	American Association of Equine Practitioners (AAEP) listserve	50	1.6
**= 8**	Dvm360	5	2.4				Google Scholar	9	1.7			
**= 8**							Merck Veterinary Manual	9	1.7			
**9**	Google Scholar	4	1.9	Veterinary Partner	36	1.6	Centers for Disease Control (CDC)	6	1.1	Equine Clinicians Network (ECN)	47	1.5
**= 9**	Medline	4	1.9				World Health Organisation (WHO)	6	1.1			
**= 9**	Science Direct	4	1.9									
**10**	5 electronic resources (3 nominations each)*	3	1.4	Cornell UCVM^$^	35	1.5	CABI or CAB abstracts Vetmed Resource	5	1.0	Google scholar	46	1.5
**= 10**		3	1.4	Equine Clinicians Network (ECN)	35	1.5	The Pig Site	5	1.0	Veterinary Partner	46	1.5
**= 10**		3	1.4				United States Department of Agriculture (USDA)	5	1.0			

^@^511 of 523 (97.7%) responses where country was not stated were responses by non-clinicians. Non-clinicians were not asked country of work in the questionnaire

*CABI or CAB abstracts, Vetmed Resource, OIE, Scopus, The Pig Site;

^$^Consultant, Dr King's Pathology or Feline Health Centre.

**Table 7:** Top ten electronic resources most accessed as nominated by clinicians and non-clinicians. Respondents could nominate up to 10 electronic resources.

Rank	Clinicians	n	%	Non-Clinicians	n	%
	(2536 responses)			(511 responses)		
**1**	VIN	649	25.6	PubMed	38	7.4
**2**	IVIS	173	6.8	University Websites or library	24	4.7
**3**	PubMed	137	5.4	OIE	23	4.5
**4**	University Websites or library	97	3.8	VIN	22	4.3
**5**	Dvm360	62	2.4	IVIS	18	3.5
**6**	American Veterinary Medical Association (AVMA)	60	2.4	American Veterinary Medical Association (AVMA)	13	2.5
**= 6**				ProMed	13	2.5
**7**	American Association of Equine Practitioners (AAEP) listserve	50	2.0	Food and Agriculture Organisation	11	2.2
**= 7**				Google	11	2.2
**8**	Google	49	1.9	Cornell UCVM^$^	9	1.8
**= 8**				Google scholar	9	1.8
**= 8**				Merck Veterinary Manual	9	1.8
**9**	Merck Veterinary Manual	38	1.5	Centers for Disease Control (CDC)	6	1.2
**= 9**				World Health Organisation (WHO)	6	1.2
**10**	4 electronic resources (37 nominations each)*	37	1.5	CABI or CAB Abstracts Vetmed Resource	5	1.0
**= 10**		37	1.5	United States Department of Agriculture (USDA)	5	1.0

^$^Consultant, Dr King's Pathology or Feline Health Centre

*Equine Clinicians Network (ECN), Google scholar, Veterinary Partner, Cornell UCVM^$^.

#### Journal and electronic resources that respondents nominated as the most useful for obtaining veterinary information

A broad range of journals (149 journals, 1159 respondents) and electronic resources (169 e-resources, 1117 respondents) were nominated as those that respondents found to be the most useful for obtaining veterinary information. There were an additional 96 responses which were discounted as they were deemed not valid (e.g. the respondent declared that they did not use one journal (n = 54) or electronic resource (n = 14) over another, or mentioned a source that was not a journal or an electronic resource).

The top ten most nominated journals accounted for more than half of the replies (643; 55.5% of 1159 respondents; Table 8). The journals with the top three nominations for being most useful were JAVMA (n = 152/1159, 13.1%), Clinician’s Brief (n = 87/1159, 7.5%) and Compendium (n = 64/1159, 5.5%) and this was also true of clinicians as a wider group. When compared to clinicians, there was also a difference in the top three journals that non-clinicians nominated that they found the most useful: Preventive Veterinary Medicine (n = 26/160, 16.3%), Veterinary Pathology (n = 23/160, 14.4%) and the Veterinary Record (n = 10/160, 6.3%; S2 Table). There was some difference between the top three journals for clinicians working in developing countries (S1 Table).

**Table 8:** Top 10 journals nominated by respondents as most useful (n = 1159).

Rank	Journal or magazine	Respondents	%
**1**	Journal of the American Veterinary Medical Association (JAVMA)	152	13.1
**2**	Clinician's Brief	87	7.5
**3**	Compendium: Continuing Education for Veterinarians	64	5.5
**4**	Journal of Veterinary Internal Medicine	58	5.0
**5**	Veterinary Medicine	54	4.7
**6**	Equine Veterinary Education	45	3.9
**7**	Equine Veterinary Journal	38	3.3
**8**	Journal of Feline Medicine and Surgery	33	2.8
**9**	Journal of Veterinary Emergency and Critical Care	29	2.5
**= 9**	Preventive Veterinary Medicine	29	2.5
**10**	Australian Veterinary Journal	27	2.3
**= 10**	Svensk Veterinärtidning	27	2.3

The electronic resources that all respondents (Table 9), and the subset of clinicians in developed countries (S3 Table), nominated as most useful were VIN, IVIS and PubMed. In developing countries, clinicians nominated similar resources as most useful in a slightly different order (IVIS, n = 16/72, 22.2%; VIN, n = 10/72, 13.9%; Google, equally placed with PubMed, n = 6/72, 8.3%; S3 Table). There were also similarities with the three electronic resources that non-clinicians found the most useful (PubMed, n = 21, 16.7%; VIN, n = 12, 9.5%; IVIS, placed equally with the World Organisation for Animal Health (OIE) n = 9, 7.1%) when compared to those nominated by clinicians (S4 Table).

**Table 9:** Top 10 Electronic resources nominated by respondents as most useful (n = 1117).

Rank	Electronic Resource	Respondents	%
**1**	VIN	533	47.7
**2**	IVIS	90	8.1
**3**	PubMed	75	6.7
**4**	Google	26	2.3
**5**	Journal of the American Veterinary Medical Association (JAVMA)	23	2.1
**6**	American Association of Equine Practitioners (AAEP) listserve	14	1.3
**= 6**	Equine Clinicians Network (ECN)	14	1.3
**= 6**	Google Scholar	14	1.3
**7**	American Association of Bovine Practitioners (AABP) listserve	13	1.2
**= 7**	Merck Veterinary Manual	13	1.2
**8**	SVA (Swedish National Veterinary Institute OR Singapore Veterinary Association)	12	1.1
**9**	University websites or library	11	1.0
**10**	OIE website	10	0.9

### Reading of scientific papers

**Fig 2:**
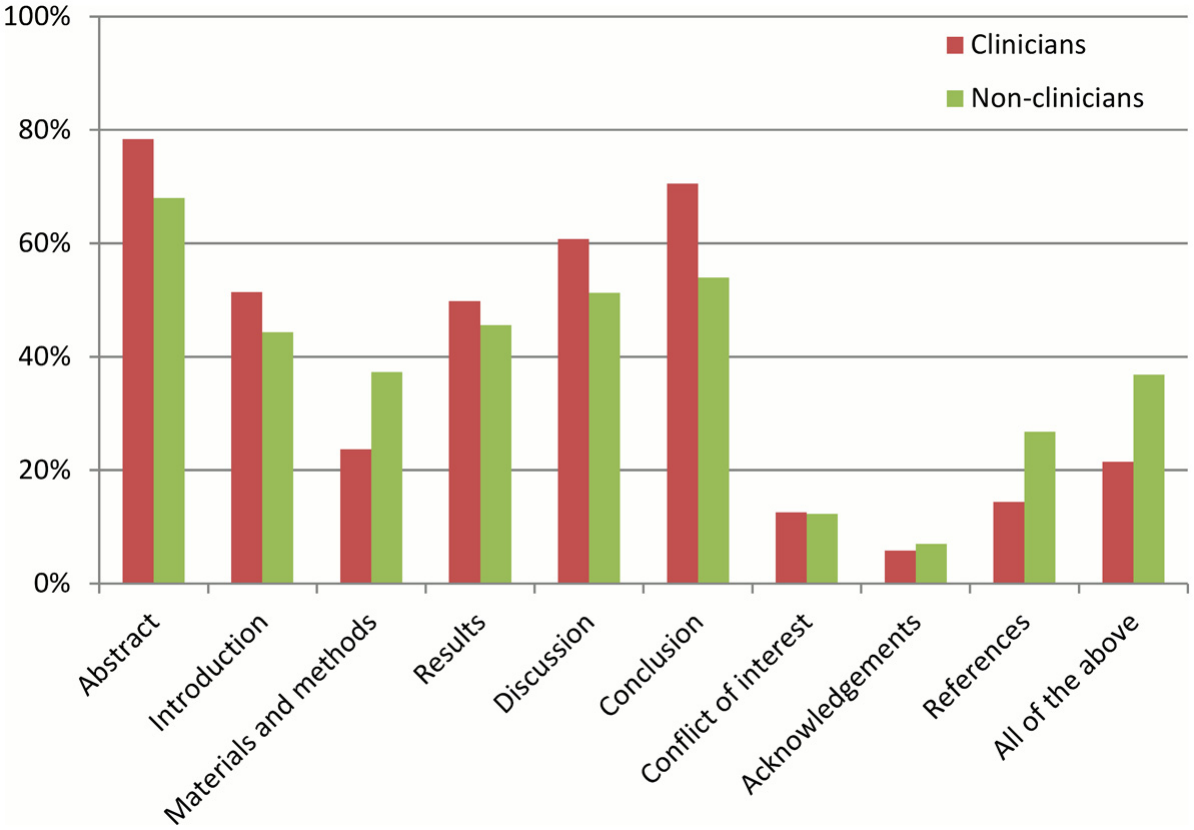
Sections of peer–reviewed paper read by clinicians and non-clinicians.

Overall, 1448 respondents (90.0% of 1609 answering the question) declared that they read peer–reviewed scientific papers. The parts of papers nominated as most commonly read were the abstract (n = 1111/1448, 76.7%), conclusions (n = 983/1448, 67.9%) and discussion (n = 858/1448, 59.3%) sections and nominated as the least read were the acknowledgements (n = 86/1448, 6.0%), conflict of interest (n = 181/1448, 12.5%) and the reference (n = 237/1448, 16.4%) sections. Non-clinicians (n = 85/228 respondents; 37.3%) reported they were more likely to read the materials and methods and the reference sections than clinicians (n = 289/1220 respondents; 23.4%; p<0.001). Non-clinicians (n = 61/228; 26.8%) were also more likely to read the reference sections than clinicians (n = 176/1220; 14.4%; p<0.001; Fig 2). The proportion of clinicians reading each section of a paper was similar between developed and developing countries.

## Discussion

This is the first study to report the types of resources used, and those which are perceived as most useful, by veterinarians internationally. There was a wide range of journal or electronic resources read by veterinarians worldwide, suggesting that there is no “one size fits all” resource for veterinary information across the globe. This has implications for EVM as, whilst some resources were clearly preferred, at the time of the survey there was no single obvious resource which veterinarians turned to in order to access the peer-reviewed evidence-base and no single place for researchers to deliver their findings. Additionally, just over 30% of respondents did not list any journals or electronic resources that they used. They may have chosen not to respond to these questions (use of open boxes instead of closed questions), may use other types of resources (e.g. textbooks) or may not regularly update themselves by accessing these resources. If the reality is the latter, there are a proportion of veterinarians not updating themselves with recent findings which has implications for the utilisation of research into practice.

The heterogeneity of resources used by respondents reflects the reality of the availability of over 1,139 journals with significant veterinary related content [24] and the large number of electronic resources available. This is pertinent for the broad ranging information requirements of those working across the different sectors of the profession. Clinicians are likely to have different information needs from non-clinicians and this was observed in the study, albeit with some overlap between groups. This was exemplified in a different study where over half of clinical questions suggested by veterinarians specifically concerned treatments [26]; such questions may not be of relevance to non-clinicians, depending on the subject matter on which they work. Despite the heterogeneity of resources named by respondents, there were a number of resources that were clearly favoured amongst the groups, both for those that were most frequently accessed and those that were deemed the most useful. For clinicians, there appeared to be some reliance on passively acquired resources and this was the case for electronic resources in particular. Passively acquired resources may be considered as those where the reader obtains information supplied directly to them in a summarised format, such as via a bulletin or newsletter. The electronic resources found to be most useful by clinicians in developed countries (VIN) and developing countries (IVIS) send out regular electronic newsletters and may be considered somewhat passively acquired whereas the favoured resource amongst non-clinicians (PubMed) potentially requires a more active approach to find information. However, VIN and IVIS and many of the other preferred electronic resources also have vast content available on their websites which is searchable and therefore active input by the reader is also an option. It may be that for busy clinicians, such sites permit active and passive strategies. Therefore, there is potentially an opportunity for the promotion of easily accessible resources that provide succinct summaries of best evidence. Banfield CATs (www.banfield.com/veterinary-professionals/resources/research/cats) and the launch of BestBETs for Vets (www.bestbetsforvets.org)[27] since this survey was conducted are two such resources that aim to fill that gap. An apparent preference for passively acquired information may also be due to other factors, such as cost-free accessibility, ease of availability or receipt of resource as part of an existing subscription, although some resources (e.g. BestBETs for Vets) are freely available. These factors may have led to an increased frequency of reporting of access of a particular resource even though it may not be the one that the reader finds the most useful. The resources that dominated amongst clinicians also tended to be those that provide clinician opinion and promote discussion, sometimes around case reports. Clinicians may relate to clinical opinion commentaries or feel that case reports are of immediate relevance to them. However, such sources do not control for bias and confounding and may or may not be evidence-based [28].

As well as time taken to access resources, accessibility may also play a role in influencing which resources are used. For example, there were some differences between the journals and electronic resources that were read by clinicians in developing countries and those in developed countries. This is not surprising since clinicians in different areas of the world may have different caseloads according to the species they see and regional disease challenges. However, access to bibliographic databases may vary and this could potentially have had an impact on the journals that respondents nominated. Twenty years ago there was poorer access to online and digital resources in developing countries [4] and it is probable that some discrepancies between the developed and developing countries remain. Another reason for this difference may be because those in developing countries were more likely to work in more than one type of work category than those in developed countries and therefore may have had wider knowledge requirements.

A large proportion of respondents to the survey were clinicians based in the USA. The clear preferences across all respondents, in particular amongst clinicians, for JAVMA and VIN may be a reflection of this respondent base. Most information available on VIN appears to be aimed at clinicians, particularly those in companion animal medicine, who also formed the majority of our respondents. One in four clinicians had heard of the survey through a veterinary website, forum, or online newsletter; this may indicate that the recruitment of respondents through VIN may have introduced a respondent bias to the electronic resource results. The work structure and percentage of respondents doing clinical work was fairly similar to those observed in recent surveys of the UK veterinary profession [15, 29].

Although the results of the survey suggest that different groups of veterinarians across the world have different information preferences it appears they have broadly similar information seeking behaviour with the majority of both groups accessing three or four journals and one electronic resource. The lower diversity of electronic resource use may be because there are fewer electronic resources, which often cover a greater scope of material than journals or that there is less awareness of electronic resources that are freely available. In the survey of the UK veterinary profession undertaken in the same year [14], a small number of resources were also found to predominate for the majority of readers. However, the most commonly accessed journal and electronic resources nominated by veterinarians outside of the UK were different to those nominated by UK veterinarians.

When reading a peer-reviewed journal article, clinicians were more likely to read the abstract and conclusions whereas non-clinicians were more likely to read the materials and methods section and the references. This is in contrast to the findings of Nielsen, Dean [14] that clinicians and non-clinicians were equally likely to read the abstract and conclusion sections. This may also support the notion that clinicians prefer to read the sections that succinctly provide ‘the answer’ to the question posed in the paper. This discrepancy between clinicians and non-clinicians may be due to the time constraints of the busy clinical role, since the time required to adequately assess the scientific information available can be a barrier [28]. It may also be due to the different emphasis on information needs between clinicians and non-clinicians particularly because the non-clinician respondents were likely to be made up of a high proportion of researchers. Researchers may be more likely to have an appreciation of a studies’ methodology as the pivotal section upon which the rest of the paper’s usefulness is assessed. Accessibility to all sections of the paper may be another explanation. Accessibility to full papers is less likely to be a constraint for non-clinicians as this is often facilitated via subscriptions by the libraries of universities and research institutes. Another reason for the differences in information seeking behaviour of clinicians and non-clinicians could be the training required to undertake thorough literature searching and appraisal. Almost twice as many non-clinicians than clinicians held a further veterinary qualification in addition to their veterinary degrees. Whilst some newer graduates may be increasingly learning literature searching and critical appraisal skills at the undergraduate level, for many these skills may not have necessarily been acquired unless doing further specialist study where official critique of the literature forms part of training.

It was not possible to know how many veterinarians received the survey and what proportion replied because of the snowball nature of the approach. The number of veterinarians in the world is unknown; it has been estimated that there are at least 500,000 veterinarians internationally [J Edwards, World Veterinary Association, personal communication. It is also therefore not possible to comment on the relative merit of each method by which veterinarians received the survey. However, the biggest source of how respondents heard about the survey was via veterinary organisations. Clearly these avenues are an important option for anyone wishing to reach the veterinary community. Organisations that are national regulatory bodies or non-regulatory organisations such as VIN, where members of a network subscribe to an email list-serve or receive regular newsletters, may therefore act as important routes to effectively disseminate key EVM messages and to communicate findings from research to the intended groups of veterinarians across the globe.

Other study limitations not already discussed include low numbers of respondents from developing countries leading to difficulties in making robust comparisons between clinicians in developing and developed countries. These low respondent numbers could be as a result of the questionnaire only being available in English, thus discouraging or excluding non-English speaking respondents. Further work focusing on information seeking behaviours of veterinarians within developing countries would be of benefit.

## Conclusion

Although there have been some studies on the use of information by veterinarians in the UK [14, 30, 31] and the United States [32], there have been no studies on the use of veterinary information sources of veterinarians globally. The results of this international survey have given us insight into the information seeking behaviour of veterinarians across the world. A wide array of journals and electronic resources are accessed by veterinarians worldwide and veterinary organisations appear to play an important role in global dissemination of information to veterinary practitioners. Clinicians in practice are likely to need information that is easily accessible and is in a summarised format for use in a timely manner, as it is possible they could adopt a more passive approach to acquiring information than non-clinicians. Further work should focus on whether access to information sources is a barrier and how the information acquired is integrated into practice by veterinarians, to further facilitate the application of EVM principles into practice.

## Supporting Information

S1 Spreadsheet Microsoft Excel spreadsheet (xlsx file) containing project data. (XLSX)

S1 Table The top ten ranked journal resources nominated as the most useful by respondents (n = 1159). Only journals with more than 1 respondent nominating them have been listed.
(DOCX)

S2 Table Top ten journals nominated as the most useful by clinicians and non-clinicians (n = 1159). Only journals with more than 1 respondent nominating them have been listed.
(DOCX)

S3 Table The top ten electronic resources nominated as the most useful by respondents (n = 1117). Only electronic resources with more than 1 respondent nominating them have been listed.
(DOCX)

S4 Table Top ten electronic resources nominated as the most useful by clinicians and non-clinicians (n = 1117). Only electronic resources with more than 1 respondent nominating them have been listed.
(DOCX)
