# Supplementary material for: International Evidence-Based Medicine Survey of the Veterinary Profession: Information Sources Used by Veterinarians
Source: PLoS One. 2016 Jul 26;11(7):e0159732. doi: 10.1371/journal.pone.0159732 (PMC4961404; doi:10.1371/journal.pone.0159732)
Supplement: S1 Table — Only journals with more than 1 respondent nominating them have been listed. (DOCX) [file pone.0159732.s002.docx]

| Rank | Developing | n | % | Developed | n | % | Country not stated | n | % | Overall | n | % |
| --- | --- | --- | --- | --- | --- | --- | --- | --- | --- | --- | --- | --- |
|  | (77 responses) |  |  | (916 responses) |  |  | (166 responses)^@^ |  |  | (1159 responses) |  |  |
| 1 | Journal of the South African Veterinary Association | 8 | 10.4 | Journal of the American Veterinary Medical Association | 139 | 15.2 | Preventive Veterinary Medicine | 26 | 15.7 | Journal of the American Veterinary Medical Association | 152 | 13.1 |
| =1 | Veterinary Medicine | 8 | 10.4 |  |  |  | Veterinary Pathology | 23 | 13.9 |  |  |  |
| 2 | Compendium: Continuing Education for Veterinarians | 6 | 7.8 | Clinician's Brief | 84 | 9.2 | Veterinary Record | 10 | 6.0 | Clinician's Brief | 87 | 7.5 |
| 3 | Vet News | 5 | 6.5 | Compendium: Continuing Education for Veterinarians | 58 | 6.3 | Journal of the American Veterinary Medical Association | 9 | 5.4 | Compendium: Continuing Education for Veterinarians | 64 | 5.5 |
| 4 | Equine Veterinary Education | 4 | 5.2 | Journal of Veterinary Internal Medicine | 47 | 5.1 |  |  |  | Journal of Veterinary Internal Medicine | 58 | 5.0 |
| =4 | In Practice | 4 | 5.2 |  |  |  |  |  |  |  |  |  |
| =4 | Journal of the American Veterinary Medical Association | 4 | 5.2 |  |  |  |  |  |  |  |  |  |
| =4 | South African Veterinary Association newsletter | 4 | 5.2 |  |  |  |  |  |  |  |  |  |
| 5 | Journal of Veterinary Internal Medicine | 3 | 3.9 | Veterinary Medicine | 46 | 5.0 | Journal of Veterinary Internal Medicine | 8 | 4.8 | Veterinary Medicine | 54 | 4.7 |
| =5 | Veterinary Record | 3 | 3.9 |  |  |  | Veterinary Microbiology | 8 | 4.8 |  |  |  |
| 6 | Avian Diseases | 2 | 2.6 | Equine Veterinary Education | 40 | 4.4 | Toxicologic Pathology | 4 | 2.4 | Equine Veterinary Education | 45 | 3.9 |
| =6 | Veterinary Clinics of North America | 2 | 2.6 |  |  |  |  |  |  |  |  |  |
| 7 | 24 journals (1 nomination each) | 1 | 1.3 | Equine Veterinary Journal | 36 | 3.9 | Clinician's Brief | 3 | 1.8 | Equine Veterinary Journal | 38 | 3.3 |
|  |  |  |  |  |  |  | Journal of Veterinary Diagnostic Investigation | 3 | 1.8 |  |  |  |
|  |  |  |  |  |  |  | Veterinary Clinics of North America | 3 | 1.8 |  |  |  |
| 8 |  |  |  | Journal of Feline Medicine and Surgery | 31 | 3.4 | 12 journals (2 nominations each)* | 2 | 1.2 | Journal of Feline Medicine and Surgery | 33 | 2.8 |
| 9 |  |  |  | Journal of Veterinary Emergency and Critical Care | 29 | 3.2 | 45 journals (1 nomination each) | 1 | 0.6 | Journal of Veterinary Emergency and Critical Care | 29 | 2.5 |
|  |  |  |  |  |  |  |  |  |  | Preventive Veterinary Medicine | 29 | 2.5 |
| 10 |  |  |  | Australian Veterinary Journal | 26 | 2.8 |  |  |  | Australian Veterinary Journal | 27 | 2.3 |
|  |  |  |  |  |  |  |  |  |  | Svensk Veterinärtidning | 27 | 2.3 |

^@^160 of 166 (96.4%) responses where country was not stated were responses by non-clinicians. Non-clinicians were not asked country of work in the questionnaire * Canadian Veterinary Journal, Journal of Dairy Science, Journal of Feline Medicine and Surgery, Journal of Veterinary Pharmacology and Therapeutics, Laboratory Animals, OIE Scientific and Technical Review, Svensk Veterinärtidning, The Veterinary Journal, Veterinary Parasitology, Veterinary World, Vetscript, Zoonoses and Public Health
