# Supplementary material for: International Evidence-Based Medicine Survey of the Veterinary Profession: Information Sources Used by Veterinarians
Source: PLoS One. 2016 Jul 26;11(7):e0159732. doi: 10.1371/journal.pone.0159732 (PMC4961404; doi:10.1371/journal.pone.0159732)
Supplement: S2 Table — Only journals with more than 1 respondent nominating them have been listed. (DOCX) [file pone.0159732.s003.docx]

| **Rank** | **Clinician** | **n** | **%** | **Non clinician** | **n** | **%** |
| --- | --- | --- | --- | --- | --- | --- |
|  | **(999 responses)** |  |  | **(160 responses)** |  |  |
| 1 | Journal of the American Veterinary Medical Association | 144 | 14.4 | Preventive Veterinary Medicine | 26 | 16.3 |
| 2 | Clinician's Brief | 84 | 8.4 | Veterinary Pathology | 23 | 14.4 |
| 3 | Compendium: Continuing Education for Veterinarians | 64 | 6.4 | Veterinary Record | 10 | 6.3 |
| 4 | Veterinary Medicine | 54 | 5.4 | Journal of the American Veterinary Medical Association | 8 | 5.0 |
| =4 |  |  |  | Veterinary Microbiology | 8 | 5.0 |
| 5 | Journal of Veterinary Internal Medicine | 51 | 5.1 | Journal of Veterinary Internal Medicine | 7 | 4.4 |
| 6 | Equine Veterinary Education | 45 | 4.5 | Toxicologic Pathology | 4 | 2.5 |
| 7 | Equine Veterinary Journal | 37 | 3.7 | Clinician's Brief | 3 | 1.9 |
| =7 |  |  |  | Journal of Veterinary Diagnostic Investigation | 3 | 1.9 |
| 8 | Journal of Feline Medicine and Surgery | 31 | 3.1 | 13 journals nominated (2 nominations each)* | 2 | 1.3 |
| 9 | Journal of Veterinary Emergency and Critical Care | 29 | 2.9 | 42 journals nominated (1 nomination  each) | | |
| 10 | Australian Veterinary Journal | 26 | 2.6 |  |  |  |

*Canadian Veterinary Journal, Journal of Dairy Science, Journal of Feline Medicine and Surgery, Journal of Veterinary Pharmacology and Therapeutics, Laboratory Animals, OIE Scientific and Technical Review, Svensk Veterinärtidning, The Veterinary Journal, Veterinary Clinics of North America, Veterinary Parasitology, Veterinary World, Vetscript, Zoonoses and Public Health
