# Supplementary material for: International Evidence-Based Medicine Survey of the Veterinary Profession: Information Sources Used by Veterinarians
Source: PLoS One. 2016 Jul 26;11(7):e0159732. doi: 10.1371/journal.pone.0159732 (PMC4961404; doi:10.1371/journal.pone.0159732)
Supplement: S3 Table — Only electronic resources with more than 1 respondent nominating them have been listed. (DOCX) [file pone.0159732.s004.docx]

| Rank | Developing | n | % | Developed | n | % | Country not stated | n | % | Overall | n | % |
| --- | --- | --- | --- | --- | --- | --- | --- | --- | --- | --- | --- | --- |
|  | (72 responses) |  |  | (915 responses) |  |  | (130 responses)^@^ |  |  | (1117 responses) |  |  |
| 1 | IVIS | 16 | 22.2 | VIN | 510 | 55.7 | PubMed | 23 | 17.7 | VIN | 533 | 47.7 |
| 2 | VIN | 10 | 13.9 | IVIS | 65 | 7.1 | VIN | 13 | 10.0 | IVIS | 90 | 8.1 |
| 3 | Google | 6 | 8.3 | PubMed | 46 | 5.0 | IVIS | 9 | 6.9 | PubMed | 75 | 6.7 |
| =3 | PubMed | 6 | 8.3 |  |  |  | OIE | 9 | 6.9 |  |  |  |
| 4 | dvm360 website | 2 | 2.8 | Journal of the American Veterinary Medical Association | 23 | 2.5 | Cornell UCVM (Consultant, Dr King's Pathology or Feline Health Centre) | 5 | 3.8 | Google | 26 | 2.3 |
| =4 | Google Scholar | 2 | 2.8 |  |  |  | Google | 5 | 3.8 |  |  |  |
| =4 | Merck Veterinary Manual | 2 | 2.8 |  |  |  | ProMed | 5 | 3.8 |  |  |  |
| =4 | South African Veterinary Association (SAVA) Ruralvet chat group | 2 | 2.8 |  |  |  |  |  |  |  |  |  |
| 5 | 26 journals (1 nomination each) | 1 | 1.4 | Google | 15 | 1.6 | American Veterinary Medical Association | 4 | 3.1 | Journal of the American Veterinary Medical Association | 23 | 2.1 |
| =5 |  |  |  |  |  |  | University Websites or library | 4 | 3.1 |  |  |  |
| 6 |  |  |  | American Association of Equine Practitioners (AAEP) listserve | 14 | 1.5 | 8 journals (2 nominations each)* | 2 | 1.5 | American Association of Equine Practitioners (AAEP) listserve | 14 | 1.3 |
| =6 |  |  |  | Equine Clinicians Network (ECN) | 14 | 1.5 |  |  |  | Equine Clinicians Network (ECN) | 14 | 1.3 |
| =6 |  |  |  |  |  |  |  |  |  | Google scholar | 14 | 1.3 |
| 7 |  |  |  | American Association of Bovine Practitioners (AABP) listserve | 11 | 1.2 | 37 journals (1 nomination each) | 1 | 0.8 | American Association of Bovine Practitioners (AABP) listserve | 13 | 1.2 |
|  |  |  |  | Google Scholar | 11 | 1.2 |  |  |  | Merck Veterinary Manual | 13 | 1.2 |
| 8 |  |  |  | SVA (Swedish National Veterinary Institute OR Singapore Veterinary Association) | 10 | 1.1 |  |  |  | SVA (Swedish National Veterinary Institute OR Singapore Veterinary Association) | 12 | 1.1 |
| 9 |  |  |  | Merck Veterinary Manual | 9 | 1.0 |  |  |  | University websites or library | 11 | 1.0 |
| 10 |  |  |  | University websites or library | 7 | 0.8 |  |  |  | OIE website | 10 | 0.9 |
| =10 |  |  |  | Vetstream | 7 | 0.8 |  |  |  |  |  |  |

^@^126 of 130 (96.9%) responses where country was not stated were responses by non-clinicians. Non-clinicians were not asked country of work in the questionnaire. *American Association of Bovine Practitioners (AABP) listserve, CABI or CAB abstracts Vetmed Resource, European Food Safety Authority (EFSA), Merck Veterinary Manual, National Mastitis Council, SVA (Swedish National Veterinary Institute OR Singapore Veterinary Association), United States Animal Health Association (USAHA), Web of Science OR Web of Knowledge
