# Supplementary material for: International Evidence-Based Medicine Survey of the Veterinary Profession: Information Sources Used by Veterinarians
Source: PLoS One. 2016 Jul 26;11(7):e0159732. doi: 10.1371/journal.pone.0159732 (PMC4961404; doi:10.1371/journal.pone.0159732)
Supplement: S4 Table — Only electronic resources with more than 1 respondent nominating them have been listed. (DOCX) [file pone.0159732.s005.docx]

| **Rank** | **Clinician** | **n** | **%** | **Non clinician** | **n** | **%** |
| --- | --- | --- | --- | --- | --- | --- |
|  | **(991 responses)** |  |  | **(126 responses)** |  |  |
| 1 | VIN | 521 | 52.6 | PubMed | 21 | 16.7 |
| 2 | IVIS | 81 | 8.2 | VIN | 12 | 9.5 |
| 3 | PubMed | 54 | 5.4 | IVIS | 9 | 7.1 |
| =3 |  |  |  | OIE | 9 | 7.1 |
| 4 | Journal of the American Veterinary Medical Association | 23 | 2.3 | Cornell UCVM (Consultant, Dr King's Pathology or Feline Health Centre) | 5 | 4.0 |
| =4 |  |  |  | Google | 5 | 4.0 |
| =4 |  |  |  | ProMed | 5 | 4.0 |
| 5 | Google | 21 | 2.1 | American Veterinary Medical Association | 4 | 3.2 |
| =5 |  |  |  | University websites or library | 4 | 3.2 |
| 6 | American Association of Equine Practitioners (AAEP) listserve | 14 | 1.4 | 7 e resources nominated (2 nominations each)* | 2 | 1.6 |
|  | Equine Clinicians Network (ECN) | 14 | 1.4 | 38 e resources nominated (1 resource each) | 1 | 0.8 |
| 7 | Google Scholar | 13 | 1.3 |  |  |  |
| 8 | American Association of Bovine Practitioners (AABP) listserve | 12 | 1.2 |  |  |  |
| 9 | Merck Veterinary Manual | 11 | 1.1 |  |  |  |
| 10 | SVA (Swedish National Veterinary Institute OR Singapore Veterinary Association) | 10 | 1.0 |  |  |  |

*CABI or CAB abstracts, Vetmed Resource, European Food Safety Authority (EFSA), Merck Veterinary Manual, National Mastitis Council, SVA (Swedish National Veterinary Institute OR Singapore Veterinary Association), United States Animal Health Association (USAHA), Web of Science OR Web of Knowledge
